# Cannibalism and Predation as Paths for Horizontal Passage of *Wolbachia* between Terrestrial Isopods

**DOI:** 10.1371/journal.pone.0060232

**Published:** 2013-04-10

**Authors:** Winka Le Clec’h, Frédéric D. Chevalier, Lise Genty, Joanne Bertaux, Didier Bouchon, Mathieu Sicard

**Affiliations:** Laboratoire Écologie et Biologie des Interactions, équipe Écologie, Évolution, Symbiose, UMR 7267 CNRS, Bâtiment B8, Poitiers, France; International Atomic Energy Agency, Austria

## Abstract

The alpha-proteobacteria *Wolbachia* are the most widespread endosymbionts in arthropods and nematodes. Mainly maternally inherited, these so-called sex parasites have selected several strategies that increase their vertical dispersion in host populations. However, the lack of congruence between the *Wolbachia* and their host phylogenies suggests frequent horizontal transfers. One way that could be used for horizontal *Wolbachia* transfers between individuals is predation. The aim of this study was to test whether horizontal passage of *Wolbachia* is possible when an uninfected terrestrial isopod eats an infected one. After having eaten *Armadillidium vulgare* harbouring *Wolbachia,* the predator-recipients (the two woodlice *A. vulgare* and *Porcellio dilatatus dilatatus*) that were initially *Wolbachia*-free were tested positive for the presence of *Wolbachia* both by quantitative PCR and Fluorescence *in situ* Hybridization (FISH). Even if the titers were low compared to vertically infected individuals, this constitutes the first demonstration of *Wolbachia* occurrence in various organs of an initially uninfected host after eating an infected one.

## Introduction

*Wolbachia pipientis* are endocytoplasmic alpha-proteobacteria widespread among arthropods [1], [2] and filarial nematodes [3]. These symbionts are mainly maternally inherited and several extended phenotypes have been naturally selected to increase their vertical transmission through generations. The extended phenotypes of the *Wolbachia* in their hosts that lead to an increased number of infected females are cytoplasmic incompatibility [1], [4], [5], male killing [6], [7], thelytokous parthenogenesis [8]–[10] and feminization of genetic males [11]–[13]. Even if the vertical transmission corresponds to the main transmission route for *Wolbachia*, several co-phylogenetic studies have revealed a lack of congruence between host and symbiont phylogenies, showing the ability of these symbionts to jump horizontally from one host to another [14]–[22]. Experimentally, it is possible to mimic such horizontal transfers (*i.e.* infection of individuals that were born without *Wolbachia*) by injecting *Wolbachia* in asymbiotic individuals [23]–[31]. However, the paths used by *Wolbachia* to pass horizontally from one individual to another in the wild are not well understood. It is obvious that such transfers would be more likely to occur between individuals that are ecologically connected. For instance, one path for *Wolbachia* to infect a new species is the hybridization process that can occur between two host species as demonstrated in the *Nasonia* species complex [21]. Interspecific interactions such as host-parasite relationships can also open a gate for *Wolbachia* to pass from one host species to another. In this context, Huigens et al. [10], [32] have experimentally demonstrated natural inter- and intraspecific horizontal transfers of parthenogenesis-inducing *Wolbachia* between parasitoid wasps of the genus *Trichogramma* sharing a same host egg. Furthermore, Heath et al. [33] showed that *Wolbachia* could pass from *Drosophila simulans* to the parasitoid wasp *Leptopilina boulardi.* Blood contact between two injured individuals is another possible path for horizontal transfer of *Wolbachia* as experimentally demonstrated for the terrestrial crustacean *Armadillidium vulgare* [34].

Another important ecological link between hosts that can potentially be used by *Wolbachia* to pass horizontally from one host to another is their trophic connection within the food chain. Several previous studies sought to investigate the capacity of *Wolbachia* to pass between individuals of different species via predation. This has been tested for spiders [19], [35], mites [36] and for some mosquito predators that were infected by the pathogenic *Wolbachia* strain *w*Melpop [37]. Despite these efforts, no successful horizontal transfer via predation could be demonstrated to date. In one case, predators became *Wolbachia-*positive when *Phytoseiulus persimilis* mites were experimentally fed with symbiotic larvae of the spider mite *Tetranychus urticae* [36]. However, the *Wolbachia* were restricted to the predator gut and starvation sufficed to erase all trace of infection. These results as well as those obtained on ticks that were thought to be infected by *Wolbachia* but were in fact infected by endoparasitoids that hosted the symbionts [38] point out that the detection of *Wolbachia* in specimens collected from the wild is not always a proof of an actual infection of the focal species. Indeed, the detected *Wolbachia* can be restricted to the intestine containing prey during digestion or to parasites infecting this species. Testing a potential route of horizontal passage of *Wolbachia* from one individual to another thus requires experiments with lineages controlled for their infection status.

Here, we used our controlled lineages of woodlice to test whether predation and cannibalism could constitute a path for *Wolbachia* transmission between individuals. Woodlice are of interest to address this question for several reasons: First of all, even if the main diet of these crustaceans is detritivorous, they can easily turn into herbivores [39] or even carnivores. They can prey on other woodlice from the same species or from different species, especially during social interactions [40]. The probability to be eaten by congenerics or conspecifics increases for individuals that are weak, for example being injured or in the middle of a molting period [41]. Another advantage of using terrestrial isopods is their relatively long lifespan (a few years) that allows the monitoring of the infection status in the same individuals for months after the initial infection. Furthermore, their size makes it possible to test different organs separately for the presence of *Wolbachia*. The ability of woodlice to get infected by eating their congeners has previously been tested by Juchault et al. [42], who fed some uninfected *A. vulgare* with pieces of individuals infected with *Wolbachia.* They concluded that this was not a path used by *Wolbachia* as they found no infection in the recipients. However, in that case, the presence of *Wolbachia* in the recipients was only tested in ovaries by transmission electron microscopy, which offers only limited sample prospection and is thus not convenient for the detection of low *Wolbachia* quantities.

In the present study, we tested the occurrence of horizontal passages of *Wolbachia* via (i) cannibalism between two *A. vulgare,* one of which being naturally infected with *Wolbachia*, and (ii) predation where an uninfected *Porcellio dilatatus dilatatus* eats an infected *A. vulgare*. To do so, we designed an experiment that increased the probability of predation and cannibalism for the woodlice [43]: Before putting predator and prey together, infected “prey” individuals were weakened by withdrawing a large amount of hemolymph while uninfected “predator” individuals were starved. Using this approach, all “prey” individuals were eaten by “predator” individuals within two days. In the months following the predation event, the presence and quantities of *Wolbachia* in the tissues of the “predators” were assessed by quantitative PCR and Fluorescence *in situ* Hybridization. Thereby, we show for the first time the occurrence of *Wolbachia* in various organs of an initially uninfected host after eating an infected one.

## Materials and Methods

### Ethic Statement

All experimental procedures and animal manipulations did not require an ethics statement.

### Biological Materials

All the animals used in these experiments were reared at 20°C in plastic breeding boxes, in natural photoperiod, on a moistened potting mix derived from peat from sphagnum moss (pH  = 6.4 and conductivity  = 50.0 mS/m) with dead lime-tree leaves as a food source. The “predators” (*i.e.* animals eating an infected terrestrial isopod) for all experiments came from controlled asymbiotic (*i.e. Wolbachia*-free) lineages of *Armadillidium vulgare* and *Porcellio dilatatus dilatatus.* The animals used to create these asymbiotic *A. vulgare* and *P. d. dilatatus* control lineages were collected in Nice (France) in 1967 and in Rom (France) in 1988, respectively, and have since been reared in the laboratory. *Wolbachia* donors, hereafter called the “preys” (*i.e.* animals eaten by the “predators”), were *A. vulgare* infected with the *w*VulC *Wolbachia* strain, from a controlled infected lineage created with animals sampled in Niort (France) and reared in the laboratory since 1961.

### Cannibalism and Predation Experiments

Thirty asymbiotic one year-old *A. vulgare* and *P. d. dilatatus* females were starved during three months in rearing boxes with no other food source than the substrate (*i.e.* no dead lime-tree leaves). After three months, starved *A. vulgare* or *P. d. dilatatus* “predators” were individually placed in boxes with one *A. vulgare* “prey”. Before being put together with the “predator”, the *A. vulgare* “preys” were weakened by collecting 10 µL of their hemolymph after piercing their cuticle with a thin needle [44]. A fraction of the collected hemolymph was used to check the *Wolbachia* infection status of the “prey”. After 48 hours, all the “preys” had been eaten by the “predators”. “Predators” were then maintained in individual boxes with substrate and lime-tree leaves *at libitum* as a food source. For each “predator” species, animals were dissected at both 90 days and 180 days post-ingestion (PI) for DNA extraction and FISH experiments.

### Tissue Samples and DNA Extractions for *Wolbachia* Quantification

Total DNA was extracted from the hemocytes (*i.e* immune cells) collected from each “prey” and the ovaries, central nervous system (*i.e.* nerve cells and neighbouring adipocytes) and hemocytes of each “predator” after dissection at 90 days and 180 days PI as described by Kocher et al. [45]. For each sample, concentration and quality (OD ratios 260/280 nm and 260/230 nm) of the extracted DNA were measured using the Nanodrop 1000 spectrophotometer (Thermo) to remove any low quality DNA sample.

### Quantification of *Wolbachia* in Host’s Tissues by qPCR

The quantification of *Wolbachia* by quantitative PCR (qPCR) was performed on (i) 10 “predators” per species (*i.e. A. vulgare* and *P. d. dilatatus*) at t = 0 before any contact of asymbiotic animals with infected “prey”, (ii) 15 “predators” per species at 90 days PI and (iii) 13 “predators” for *P. d. dilatatus* and only two for *A. vulgare* at 180 days PI. Moreover, *Wolbachia* quantification was performed on the hemocytes collected from all 60 *A. vulgare* “prey” individuals.

qPCR reactions were performed using the LightCycler 480 system (Roche) as follows: 10 min at 95°C followed by 45 cycles [10 sec at 95°C, 10 sec at 60°C, 20 sec at 72°C]. A melting curve (65°C to 97°C) was recorded at the end of each reaction in order to check the specificity of the PCR product. The reaction mixture consisted of 5 µL of SYBRGreen MasterMix (Roche), 0.5 µL of each 10 µM specific primers [*wsp*208f (5′- TGG-TGC-AGC-ATT-TAC-TCC-AG-3′) and *wsp*413r (5′-TCG-CTT-GAT-AAG-CAA-AAC-CA-3′)], amplifying 205 bp of the single-copy *Wolbachia* surface protein (*wsp*) gene, 3 µL of sterile water and 1 µL of template DNA (between 10 ng and 80 ng of DNA). A Standard curve was plotted using seven dilutions of a purified *wsp* PCR product (*wsp* copies.µL^−1^: 2.63×10^0^, 2.63×10^1^, 2.63×10^2^, 2.63×10^3^, 2.63×10^4^, 2.63×10^5^, 2.63×10^6^, 2.63×10^7^). The number of *wsp* copies was estimated according to the standard curve. The total DNA quantity (*i.e*. host+*Wolbachia*) of each sample was used to normalize the resulting *wsp* gene copy counts. The results are thus given in number of *wsp* copies per ng of total DNA.

### *Wolbachia* Localization in “Predator” Hemocytes Using Fluorescence *in situ* Hybridization (FISH)

Hemocytes were sampled from “predators” of each species (*i.e.* two *A. vulgare* females and five *P. d. dilatatus* females) 180 days PI. Each animal yielded ∼10 µL of hemolymph that were pooled for each species in a microtube kept on ice. Fluorescence *in situ* Hybridization was performed according to Chevalier et al. [46] on a total of 4 µL for each pool fixed with 1% paraformaldehyde-PBS solution (137 mM NaCl, 8 mM Na_2_HPO_4_, 12H_2_O, 1.5 mM KH_2_PO_4_, 3 mM KCl, pH 7.3), at 35% formamide with the probes W1-Cy3 (5′-AATCCGGCCGARCCGACCC-3′) and W2-Cy3 (5′-CTTCTGTGAGTACCGTCATTATC-3′) (30 ng.mL^−1^ each) targeting *Wolbachia* 16S rRNA [47]. As in Chevalier et al. [46] the samples were further stained with FITC-phalloidin (20 ng, Sigma) mixed into the hybridization buffer and with DAPI (Sigma) mixed into the mounting medium (AF1 antifading, Citifluor, England, 2.5 mg.mL^−1^). Detection was performed with an epifluorescent microscope (Axio Observer-A1, Zeiss) with Apotome (structured illumination) equipped with a 63X/1.25 objective (oil immersion) and with the AxioVision 4.8.1 software (Zeiss). The number of hemocytes and their *Wolbachia* colonization status were counted on ten random images per pool, using ImageJ software (version 1.45; [48]). Hemocytes touching the image borders, *i.e.* incomplete ones, were ignored.

### Statistical Analysis

All statistical analyses were performed using R software (version 2.10.1). Since data distribution did not follow a normal distribution (Shapiro test, p<0.05), *Wolbachia* titers in tissues were compared with a Kruskal-Wallis followed by Dunn’s multiple comparison test or simple pairwise comparison Wilcoxon-Mann-Whitney tests.

## Results

### *Wolbachia* Quantification in the Different Host Tissues of “Predators”

**Figure 1:**
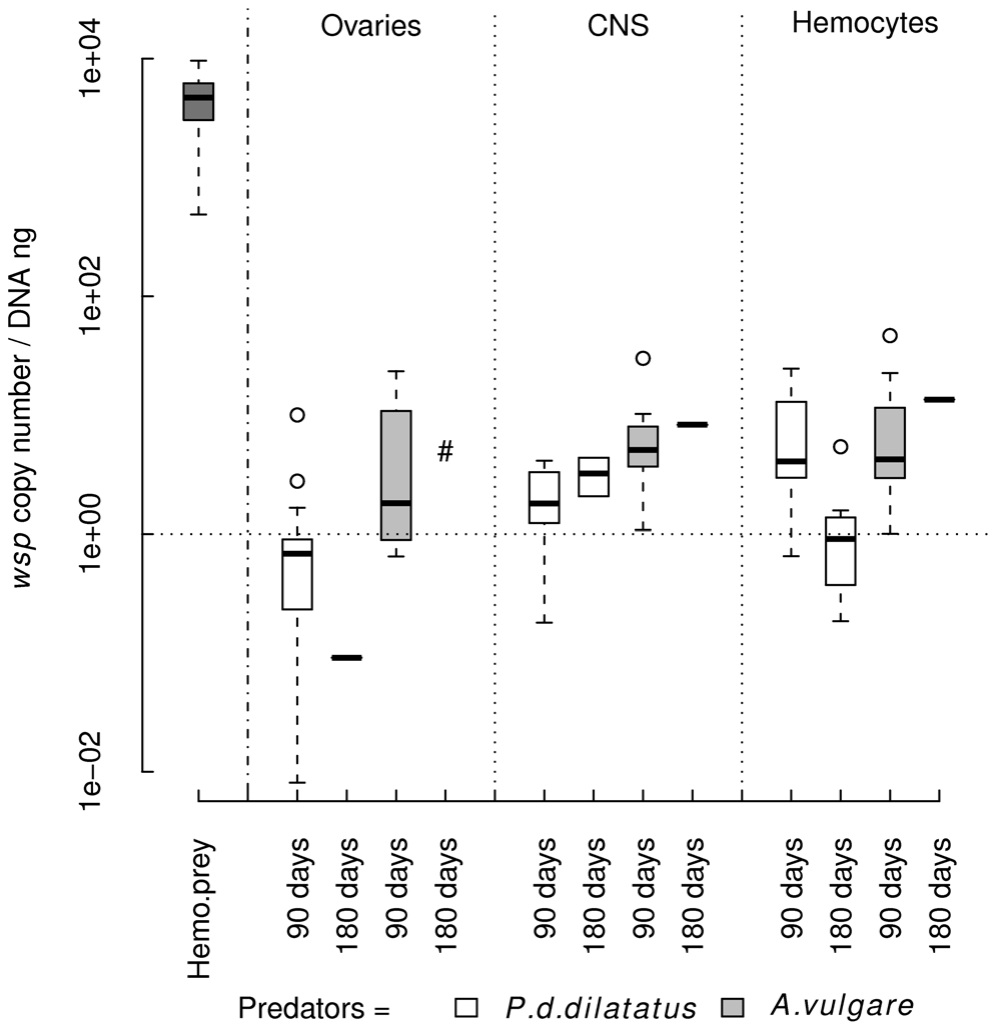
*Wolbachia* loads in CNS, ovaries and hemocytes of the “predators” *Porcellio d. dilatatus* and *Armadillidium vulgare*. The *Wolbachia* quantifications performed at 90 and 180 days post-ingestion (PI) revealed that the symbiont colonizes all tissues of the asymbiotic “predators” after ingestion of infected “preys”. Comparison of the *Wolbachia* titers between the two predator species (*i.e. P. d. dilatatus* or *A. vulgare*) revealed that bacterial loads were higher in *A. vulgare*, the native host of *w*VulC strain, at 90 days PI. Comparison of *Wolbachia* titers in the different tissues of *P. d. dilatatus* between 90 and 180 days PI showed that the infection was stable in ovaries and CNS but decreased in hemocytes. (Nat.titer: The *Wolbachia* titer in the “prey” hemolymph; #: No *A. vulgare* were infected with *Wolbachia* among the two tested animals).

The *Wolbachia* were quantified by qPCR in hemocytes of the symbiotic “preys” and in three “predator” tissues: ovaries, central nervous system (CNS) and hemocytes at 90 and 180 days post-ingestion (PI). All the “preys” harboured high titers of *Wolbachia* (mean of *Wolbachia* in hemocytes ± se/ng DNA: 4.62×10^3^ ±4.57×10^2^, Fig. 1), which is in accordance with titers previously reported in Le Clec’h et al. [49]. All the “predators” tested at t = 0 before any contact with the “preys” were completely asymbiotic since not a single *Wolbachia* was detected by qPCR in all tested tissues.

At 90 days PI, all tested “predators” from both species (*i.e. A. vulgare* and *P. d. dilatatus*) exhibited *Wolbachia* in all tested tissues but at low titers comprised between one and 100 *Wolbachia* per ng DNA (Figure 1). In both ovaries and CNS, *Wolbachia* titers were higher in *A. vulgare*, the native host of *w*VulC, than in *P. d. dilatatus* (Wilcoxon-Mann-Whitney test: U = 26; *p = *0.0147 and U = 14; *p = *0.0009, respectively), while no difference was found between species regarding *Wolbachia* titers in hemocytes (Wilcoxon-Mann-Whitney test: U = 64; *p = *0.9730). A global comparison of *Wolbachia* titers between the different tissues of *P. d. dilatatus* highlighted heterogeneity between them (Kruskal-Wallis test: K = 14.583, *df* = 2, *p* = 0.0007). This heterogeneity was due to higher titers of *Wolbachia* in hemocytes than in ovaries (Dunn Multiple comparison test: *p<*0.001). On the contrary, global comparison of *Wolbachia* titers between the different tissues of *A. vulgare* revealed no difference (Kruskal-Wallis test: K = 2.818, *df* = 2, *p* = 0.2440).

At 180 days PI, many organs were found uninfected by *Wolbachia,* suggesting an overall decrease of the infection compared to 90 days PI (Table 1). However, at this time point, most *A. vulgare* individuals were dead (Table 1). We assume that this high mortality rate was due to their isolation and the lack of social contacts that are strongly required for this species exhibiting an important gregarious lifestyle [40]. The comparison of *Wolbachia* titers in the different tissues of *P. d. dilatatus* between 90 and 180 days PI showed that the infection was stable in ovaries (Wilcoxon-Mann-Whitney test: U = 10; p = 0.1818) and CNS (Wilcoxon-Mann-Whitney test: U = 5; *p = *0.2286) but decreased in hemocytes (Wilcoxon-Mann-Whitney test: U = 11.5; *p = *0.0019; Figure 1).

**Table 1:** *Wolbachia* detection in predators 180 days after ingestion of an infected prey.

	Total	Alive	*Wolbachia* detection in ovaries	*Wolbachia* detection in CNS	*Wolbachia* detection in hemocytes
*Porcellio d. dilatatus*	15	13	4/13	13/13	13/13
*Armadillidium vulgare*	15	2	0/2	1/2	1/2

### Detection of *Wolbachia* in the “Predator” Hemocytes from Both Species Using Fluorescence *in situ* Hybridization (FISH)

**Figure 2:**
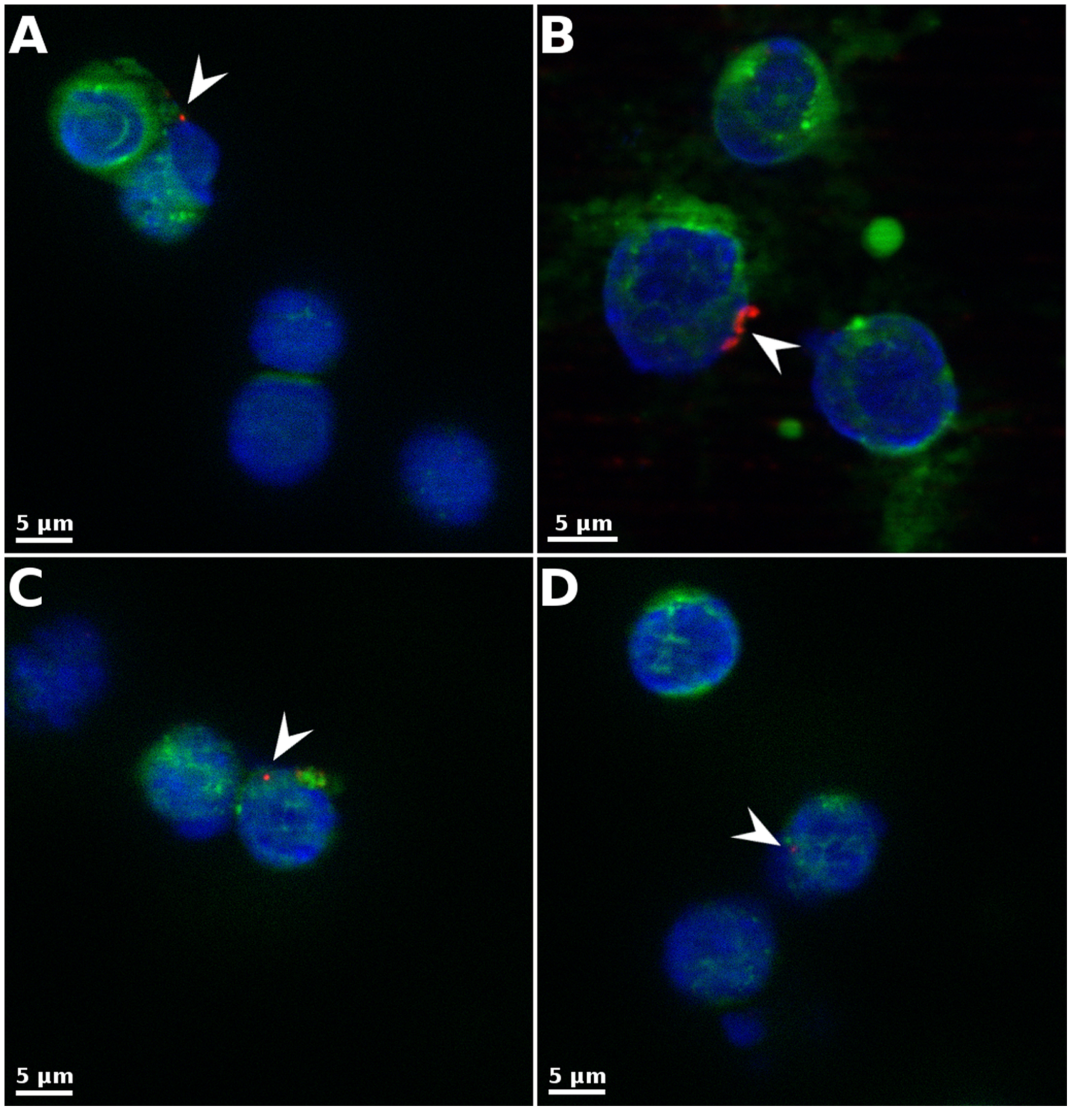
Fluorescence *in situ* Hybridization (FISH) detection of *Wolbachia* in circulating hemocytes of the “predators” *Porcellio d. dilatatus* and *Armadillidium vulgare* 180 days after having ingested of an infected prey. Some hemocytes of *P. d. dilatatus* (A and B) and of *A. vulgare* (C and D) were infected with *Wolbachia* (3 and 8% of hemocytes, respectively). A-D: *Wolbachia* in red, Actin in green, nuclei in blue.

*Wolbachia* quantification by qPCR revealed that “predators” were infected by *Wolbachia* at a low level after the ingestion of symbiotic (*i.e. Wolbachia*-positive) “preys”. This was confirmed by FISH detection of *Wolbachia* with specific probes on one pool of hemolymph per species, with two animals for *A. vulgare* and five for *P. d. dilatatus* at 180 days PI. Hemocytes of both *P. d. dilatatus* (Figures 2A and B) and *A. vulgare* (Figures 2C and D) “predators” were colonized (3 and 8%, respectively). The fact that the probes target the transcript of the 16S rRNA gene further indicates that the *Wolbachia* were alive.

## Discussion

Predation, though a brief interaction between living individuals of two species, is used by many parasites that pass horizontally from one host to another [50]. Cannibalism, which is one peculiar case of predation that occurs between individuals belonging to the same species, can also lead to horizontal parasite transmission [51]. The horizontal passage of *Wolbachia* from one individual to another by the predation route was tested unsuccessfully in several studies [36], [37]. Phylogenetic analyses of the *Wolbachia* infecting spiders and their prey in the same ecosystem also indicated that *Wolbachia* would not frequently navigate between species using this route [35]. On a different note, some Endosymbiont Based Control Strategies rely on the principle that after introduction into a new host and release in the environment, *Wolbachia* will not escape and potentially spread along the food chain through predators. Indeed, when artificially introduced into the mosquito *Aedes aegypti*, the *Wolbachia* strain *w*MelPop was not found in the five natural predator species tested after preying on larvae or adults [37]. In one case, predators became *Wolbachia-*positive when *Phytoseiulus persimilis* mites were experimentally fed with symbiotic larvae of the spider mite *Tetranychus urticae* [36]. However, the *Wolbachia* were restricted to the predator gut and starvation sufficed to erase all trace of infection, showing that, in this case, the *Wolbachia* did not cross the intestine wall.

Here, we show that after ingestion of an *A. vulgare* “prey”, *w*VulC *Wolbachia* cells were detected in organs and blood of two different “predator” species. This indicates for the first time that some *Wolbachia* were able to resist the digestive process, to pass through the intestine barrier and to infect the tested tissues (*i.e.* ovaries, CNS and hemocytes). The efficiency of such a transfer was very high since 100% of the “predators” of both species were infected by *w*VulC, even if all their tested tissues were not always infected at 180 days PI. Overall, the titers of *Wolbachia* were higher after intraspecific passages in *A. vulgare* via cannibalism than after interspecific passages in *P. d. dilatatus* via predation.

However, the *w*VulC titers in all tested tissues remained very low compared to those recorded in naturally infected *A. vulgare* or after injection of *Wolbachia* in the haemocoel of initially asymbiotic individuals of *P. d. dilatatus* or *A. vulgare* [49]. Moreover, the *Wolbachia* loads did not increase between 90 and 180 days after ingestion, suggesting very low or no multiplication. It can be hypothesized that due to the stress generated by the environment of the digestive tract, the *Wolbachia* switched to a form that does not multiply or does so at a very low rate. A first hypothesis to further explain this pattern is that only very few *Wolbachia* variants were able to cross the intestine wall and then multiplied to reach only a low density in the host. Thus, this strong bottleneck would have selected for the *Wolbachia* that are more resistant while exhibiting lower multiplication rates, according to a growth/survival trade-off [52]. A second hypothesis is that many bacteria survived the intestine, crossed the wall and colonized the tissues as a “non-replicative form” that slowly decayed over months. The ability of *Wolbachia* to produce “non-replicative forms” has not yet been closely investigated. However, it has been shown previously that *Wolbachia* is able to survive without replicating in stressful conditions like heat treatment [53], [54] or upon extracellular maintaining for as long as one week [12], [55]. At 180 days PI, we found tissues of some individuals without *Wolbachia* infection: Ovaries of some *P. d. dilatatus* and ovaries, CNS and hemocytes of some *A. vulgare*. We cannot exclude that these tissues had never been infected. However, since all tissues were infected in all animals tested at 90 days PI, we rather suspect that the bacteria died, either because of their inability to recover from the stress encountered during the horizontal passage and/or due to host reactions such as autophagy or apoptosis that are known to be involved in the elimination of *Wolbachia* from host cells [49], [56], [57].

Among all the tested tissues, the CNS presented an interesting pattern: The infection was low but seemed to be stable until 180 days PI in both species. Regardless of the transinfection mode (*i.e.* injection or ingestion), the *w*VulC strain seems to colonize the CNS in a more extensive way than the other tissues [49]. This tissue seems to constitute a kind of refuge for the *Wolbachia,* possibly because nerve cells are renewed less frequently compared to hemocytes and oocytes. However, in the nerve cells of individuals infected by the predation route, the infection was stable but low and did not trigger the pathogenic phenotype described in *P. d. dilatatus* injected with *w*VulC from *A. vulgare* ovaries [49].

In this study, we demonstrate that predation and cannibalism can lead to the ingestion of *Wolbachia* in terrestrial isopods. Moreover, following this ingestion the presence of *Wolbachia* is not restricted to the intestine as other organs were tested positive. This demonstrates that *Wolbachia w*VulC is capable of crossing the intestine barrier and to survive, at least transiently, in a predator’s body.
